# Bayesian phylogeographic analysis infers cross-border transmission dynamics of drug-resistant *Salmonella* Enteritidis

**DOI:** 10.1128/spectrum.02292-24

**Published:** 2025-02-07

**Authors:** Pei Yee Woh, Yehao Chen, Kevin Wing Hin Kwok, Jose Quiroga

**Affiliations:** 1 Department of Food Science and Nutrition, The Hong Kong Polytechnic University 26680 , Hong Kong Special Administrative Region, China; 2 Research Institute for Future Food (RiFood), The Hong Kong Polytechnic University, Hong Kong Special Administrative Region, China; 3 Office of Global Outreach and Extended Education, Ira A. Fulton Schools of Engineering, Arizona State University, Tempe, USA; University at Albany, Albany, New York, USA

**Keywords:** *Salmonella *Enteritidis, antimicrobial resistance, Bayesian phylogeography, whole genome sequencing, migration

## Abstract

*Salmonella* Enteritidis (*S*. Enteritidis) stands as a leading cause of human salmonellosis worldwide with a tendency to spread through contaminated foodstuffs and animals. In Hong Kong, a significant proportion of food products are imported, and many cases are often caused by the consumption of contaminated food, hence making the geographical surveillance of drug-resistant *S*. Enteritidis important for strong public health and food safety measures. We analyzed the whole genomes of 207 *S*. Enteritidis from Hong Kong, Australia, Canada, mainland China, the United States of America, South Africa, Taiwan, and the United Kingdom to examine associated antimicrobial resistance and the transmission dynamics between continents. Phylogenetic cluster inferences and Bayesian phylogeographical analysis were performed. Overall, sequence type ST11 strains were dominant (92.8%, 192/207). Five phylogenomic clusters A to E were identified, where most isolates from mainland China and Hong Kong were in Cluster E. Among the 22 plasmid types identified, IncX1 was dominant in the Asian isolates. Most of the virulence genes were distributed in *Salmonella* pathogenicity islands −1 and −2, with two universal virulence operons responsible for the effector delivery system and bacterial cell adhesion. The phylogeographic inference analysis showed a statistically significant link between mainland China and Hong Kong with the highest relative migration rate (relativeGeoRates mean ± standard error = 2.93 ± .07, Bayes Factor [BF] = 1285.5], with some traceable to Canada (0.61 ± 0.03, BF = 6.9) and Australia (1.02 ± 0.04, BF = 4.2). Our analysis suggests hypothetical transmission of *S*. Enteritidis and its associated antimicrobial resistance across borders.

**IMPORTANCE:**

Antimicrobial resistance and disease severity in nontyphoidal *Salmonella* have constituted a serious public health challenge worldwide. Drug-resistant *Salmonella* Enteritidis is a leading pathogen that causes human infections primarily through the consumption of contaminated food products. Previous research focuses on the whole-genome analysis of antimicrobial resistance and virulence factors in *S*. Enteritidis; however, details on how this bacterium localized, expanded, and diversified from location to location remain unknown. Our study for the first time addresses this gap by investigating the phylogeographic transmission to estimate the frequency and location of cross-border spread. By evidence-based inferred transmission, we aim to uncover novel insights into the dynamic spread of *S*. Enteritidis, revealing the route of emergence and migration. This research is crucial for enhancing our understanding of the control strategies to safeguard human health.

## INTRODUCTION

*Salmonella enterica* serovar Enteritidis (*S*. Enteritidis) is one of the most prevalent *Salmonella* serotypes responsible for gastroenteritis and foodborne outbreaks. Studies have shown that *S*. Enteritidis strains isolated from various sources, including animals and humans, exhibited antimicrobial resistance (AMR) to multiple antimicrobial agents (1–3). The resistance of *S*. Enteritidis to critical antibiotics, like third-generation cephalosporins and fluoroquinolones raises, concerns about treatment options for infections caused by these bacteria (4).

The resistance mechanisms in *S*. Enteritidis are complex and multifaceted, often involving the presence of large plasmids carrying resistance genes contributing to the dissemination of resistance among *Salmonella* strains (5, 6). The resistance trend in *S*. Enteritidis is not limited to specific regions but is a worldwide issue, as evidenced by studies conducted in countries, like Chad, Iraq, Brazil, Russia, Ghana, and China (1, 2, 7–10). The contributing factors include changes in food production and consumption practices (11, 12), the emergence of new strains with higher virulence (13, 14), and the inappropriate or excessive use of antibiotics in both human and animal healthcare (15). However, details on how this bacterium localized, expanded, and diversified from country to country remain unknown.

The integration of whole genome sequencing (WGS) with phylogenetic analysis could provide valuable insights into this pathogen’s genetic diversity, evolution, and geographical distribution. Several studies have employed WGS to investigate the phylogenetics of *S*. Enteritidis isolates from various sources, shedding light on their transmission dynamics and genetic characteristics (16–19). These analyses have revealed distinct clades and lineages within *S*. Enteritidis populations, indicating the presence of different evolutionary paths and potential associations with specific geographical regions or hosts. By examining the core genome and accessory genome of these isolates, researchers have been able to identify key genetic features associated with AMR, virulence, and host adaptation (20–22). This approach has also been instrumental in outbreak investigations, allowing for the precise tracking of strains responsible for localized outbreaks (16, 18, 23, 24).

In Hong Kong, human salmonellosis, particularly concerning antibiotic resistance, has been a subject of research. Research has identified AMR as a growing issue among *S*. Enteritidis strains with reports of resistance to fluoroquinolones, beta-lactams, and other antimicrobial agents (6, 25–28). Given that over 90% of the local food supply is imported in Hong Kong (29), there is an increased risk of *Salmonella* pathogens being introduced into the local food supply chain (30–34), contributing to the city’s vulnerability to foodborne *Salmonella* infections. To date, however, the occurrence and dynamic spread of *S*. Enteritidis between Hong Kong and the countries or regions from which it imports food remain largely unexplored.

In the present study, we compare the phylogenetic AMR of Hong Kong data with that from other countries or regions and estimate the transmission dynamics for *S*. Enteritidis based on a Bayesian phylogeographic framework.

## MATERIALS AND METHODS

### *S.* Enteritidis strain and genome selection

A total of 45 *S*. Enteritidis clinical isolates previously collected from Hong Kong in 2019 (31) were used in this study. Briefly, the DNA libraries were prepared (Riptide DNA library preparation kit; iGenomx, USA) and sequenced using the NextSeq platform (Illumina, USA) with a paired-end option. Sequence reads were demultiplexed according to the manufacturer’s instructions. Quality control (QC) of the raw reads was performed using FastQC version 0.11.9 (35), trimmed with Trimmomatic version 0.39 (36) with the default setting (QC30) and *de novo* assembled for contigs using SPAdes version 3.15.2 (37), with a minimal length of 500 bp. In addition, WGS data of clinical *S*. Enteritidis isolates from other countries were downloaded from the National Center for Biotechnology Information (NCBI) Pathogen Detection database. The selection of isolates was based on the collection period (pre- and post-2019, i.e., between 1 January 2018 and 31 December 2020), source of isolation, and availability of genome data and associated metadata. The isolates used in this analysis did not represent a specific location within an individual region.

To represent the geographic and phylogenetic diversity of *S*. Enteritidis, we selected countries and regions with significant international trading with Hong Kong assessed on the World Integrated Trade Solution (38). The initial screening included 19 countries/regions: mainland China, Taiwan, the United Kingdom, Canada, the United States of America, Australia, New Zealand, South Africa, Peru, Japan, France, Singapore, South Korea, Indonesia, Thailand, Vietnam, Chile, Brazil, and the Netherlands. Seven countries/regions were selected depending on the availability of genome data and associated metadata. Isolates from the United Kingdom, Canada, Australia, and South Africa were selected from the “Collection Date” column on the NCBI Pathogen Detection website. Due to the availability of clinical data, isolates from mainland China (39), Taiwan (40), and the United States of America (41) were selected from three previous published studies. The low quality for having a genome assembly N50 size <100,000 or a sequence coverage <30× was excluded. Finally, we compiled a total of 207 data from Hong Kong (42), Australia (*n* = 30), Canada (*n* = 30), mainland China (*n* = 22), the United States of America (*n* = 20), South Africa (*n* = 25), Taiwan (*n* = 5), and the United Kingdom (*n* = 30) ( Table S1 to S3).

### Pan-genome analysis

DNA sequences in fasta format were annotated using Prokka version 1.14.6 (43). This process generated GFF3 files as the input for pan-genome analysis and calculation using Roary version 3.13.0 (44). The matrix detailing the presence or absence of core and accessory genes was used to plot the heatmap.

### Phylogenetic SNP analysis and multi-locus sequence typing (MLST)

**Fig 1:**
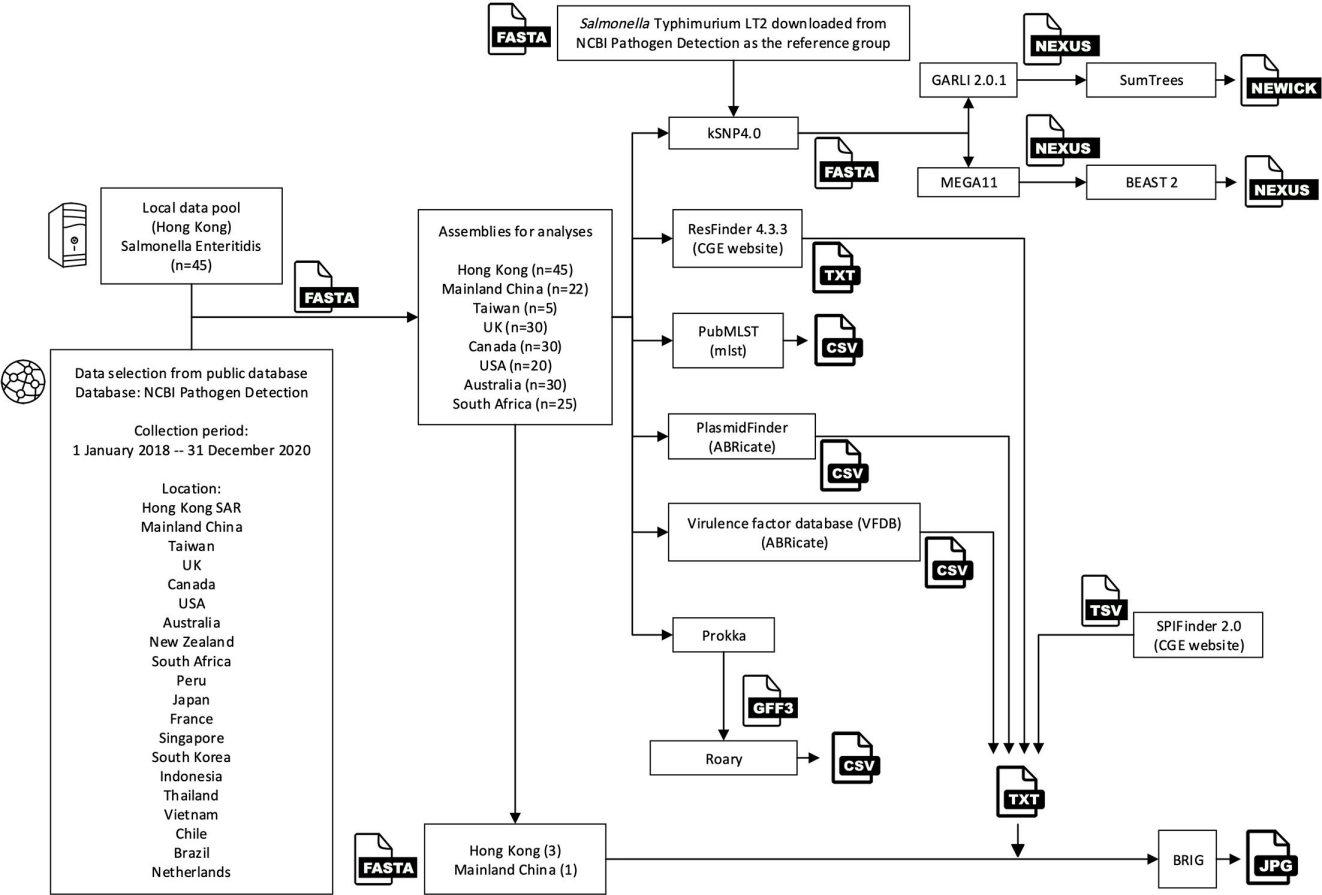
Study tools and analysis workflow.

Figure 1 illustrates the entire genomic tool and analysis workflow. To elucidate evolutionary relationships among *S*. Enteritidis isolates, *Salmonella* Typhimurium str. LT2 (NCBI bioSample accession no. SAMN15862390) was selected as a reference group to reconstruct whole-genome phylogenies. kSNP4.0 (45) with an optimum k value of 17 from Kchooser was utilized to generate a matrix of core single nucleotide polymorphisms (SNPs). Subsequently, a maximum likelihood phylogenetic tree was constructed from the core SNP alignment using GARLI2.01 (42) (ratematrix = 6 rate, ratehetmodel = gamma). Branch support for the maximum-likelihood phylogeny was assessed through a bootstrap analysis of 1,000 pseudo-replicates. Multiple runs of bootstrap analysis (*n* = 100) were performed to ensure consistent results. Python program SumTrees version 4.6.1 (46) was used to compile a consensus tree with a bootstrap value at a 70% threshold. Finally, R package ggtree version 3.8.2 (47) was used to visualize the finalized phylogenetic tree. Based on the Achtman MLST scheme, the sequence types (STs) of the studied isolates were assessed by MLST (48) against the PubMLST database (49). The STs were determined using seven housekeeping genes: *aroC, dnaN, hemD, hisD, purE, sucA,* and *thrA*.

### Antimicrobial resistance genes, plasmid replicon markers, and virulence factors

To evaluate the AMR genotype profiles of the studied isolates, we used ResFinder version 4.3.3 (50), accessible through the Center for Genomic Epidemiology (CGE) website. This online tool helped identify the presence and type of AMR genes associated with specific phenotypes, using default settings of a 90% identity threshold and 60% minimum sequence length coverage for nucleotide sequence. Isolates were classified as multidrug resistant (MDR) if they exhibited resistance to at least one agent in three or more antibiotic classes (51). Additionally, we performed a mass screening for plasmid replicons and virulence factors using ABRicate version 1.0.1 (52) against PlasmidFinder (53) and the Virulence Factors of Pathogen Bacteria Database (54), respectively. Heatmaps illustrating the presence and absence of AMR genes, plasmid replicon markers, and virulence factors in each isolate were created using R package ggtree version 3.8.2 (47).

### Bayesian phylogeographic inference analysis

The core SNP matrix (25,780 nucleotides) in fasta format generated from kSNP4.0 (45) was converted into a nexus format using MEGA11 version 11.0.13 (55). This nexus file served as the input for Bayesian phylogeographic inference analysis conducted in BEAST 2 version 2.7.5 (56). In BEAST 2, we selected the gamma site model, considered the sampling location as a discrete trait, and used the year of isolation as sampling times (tip dates). For the substitution model, HKY was selected as the site model, and empirical as the frequency model. A strict clock model and the constant population coalescent tree prior were used. With this model combination above, we performed three independent runs to ensure convergence. Trees and log files generated from these three runs were then combined using LogCombiner (56), and we ensured that the effective sample size of all parameters was at least 250 using Tracer version 1.7.2 (57). The relative rates of *S*. Enteritidis migration between countries or regions were inferred and recorded in the combined log file. In the log file, the mean of each “rateIndicator” variable represents the posterior probability that a specific transition rate is positive, and the “relativeGeoRates” variable refers to the relative migration rate between a pair of regions. This log file was further used for Bayes factors (BF) calculation via SPREAD (58), and migration events with BF values between 3 and 10 were considered moderate, and those greater than 10 were considered strong evidence (59). The output trees were summarized as a maximum-clade credibility tree using TreeAnnotator (56), followed by tree visualization in FigTree version 1.4.4 (60). The internodal branches were colored by sampling location, and the branch width was adjusted by the posterior probability. By visually inspecting the collapsed phylogenetic tree, we interpreted the directionality and number of the statistically significant migration events with BF greater than 3 and visualized the inferred migration on a geographical map.

## RESULTS

### Human-associated *S*. Enteritidis are largely represented by five major clusters

**Fig 2:**
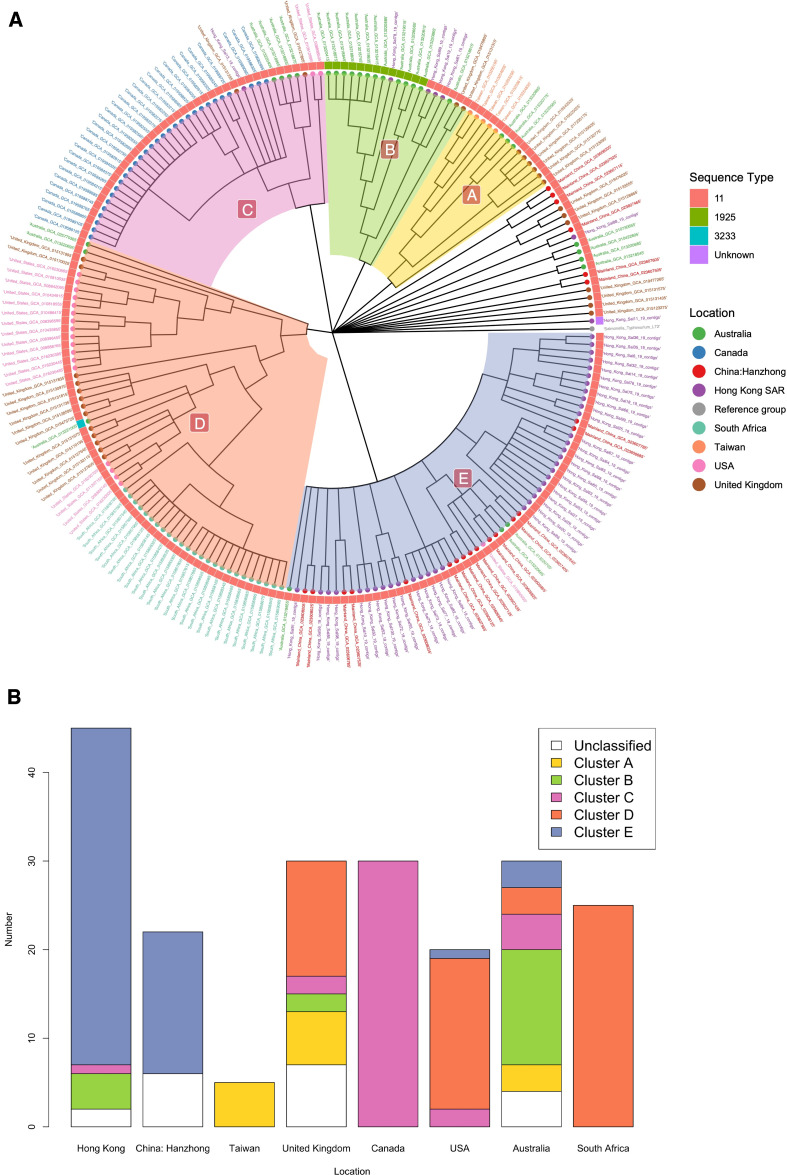
Population structure of the 207 *S*. Enteritidis isolates. (A) Maximum likelihood phylogenetic tree inferred from a complex-wide alignment of 25,780 core SNPs identified in the analyzed 207 *S*. Enteritidis isolates. The tree is rooted using *Salmonella* Typhimurium str. LT2 (NCBI BioSample accession no. SAMN15862390) as the reference genome. (B) Stacked bar chart showing the distribution of the *S*. Enteritidis isolates by geographical origin.

We identified a total of 9,390 genes that comprise the pan-genome ( Table S4 and S4a). Of these, the core genes, representing 44.7% of the pan-genome, including 4,121 genes (present in ≥99% isolates) and 78 soft-core genes (present in 95% ≤ isolates < 99%). The accessory genome, which made up 55.3% of the pan-genome, consisted of 390 shell genes (present in 15% ≤ isolates < 95%) and 4,801 cloud genes (present in <15% of isolates). The core SNP matrix, composed of 25,780 nucleotides, was used to reconstruct the phylogenetic tree from these 207 *S*. Enteritidis genomes with *S*. Typhimurium str. LT2 served as the reference genome. We identified three distinct sequence types: ST11 (92.8%, 192/207), ST1925 (6.3%, 13/207), and ST3233 (Table S5). The remaining isolate (i.e., Hong_Kong_Sal11_19_contigs) was unsubtypable. Five phylogenetically major clusters A–E were defined within these isolates (Fig. 2A). The majority of isolates collected from Hong Kong and mainland China were grouped in Cluster E. Cluster A was strongly associated with the Taiwan isolates, all of which were ST11. Despite being on the same continent, isolates from the United States (clusters C, D, and E) and Canada exhibited distinct patterns (Fig. 2B). Isolates from Canada and South Africa exclusively belonged to clusters C and D, respectively. In contrast, Australia and the United Kingdom exhibited a geographically diverse and cosmopolitan collection of isolates, spanning all five clusters A to E.

### Antimicrobial resistance of *S*. Enteritidis varies geographically

**Fig 3:**
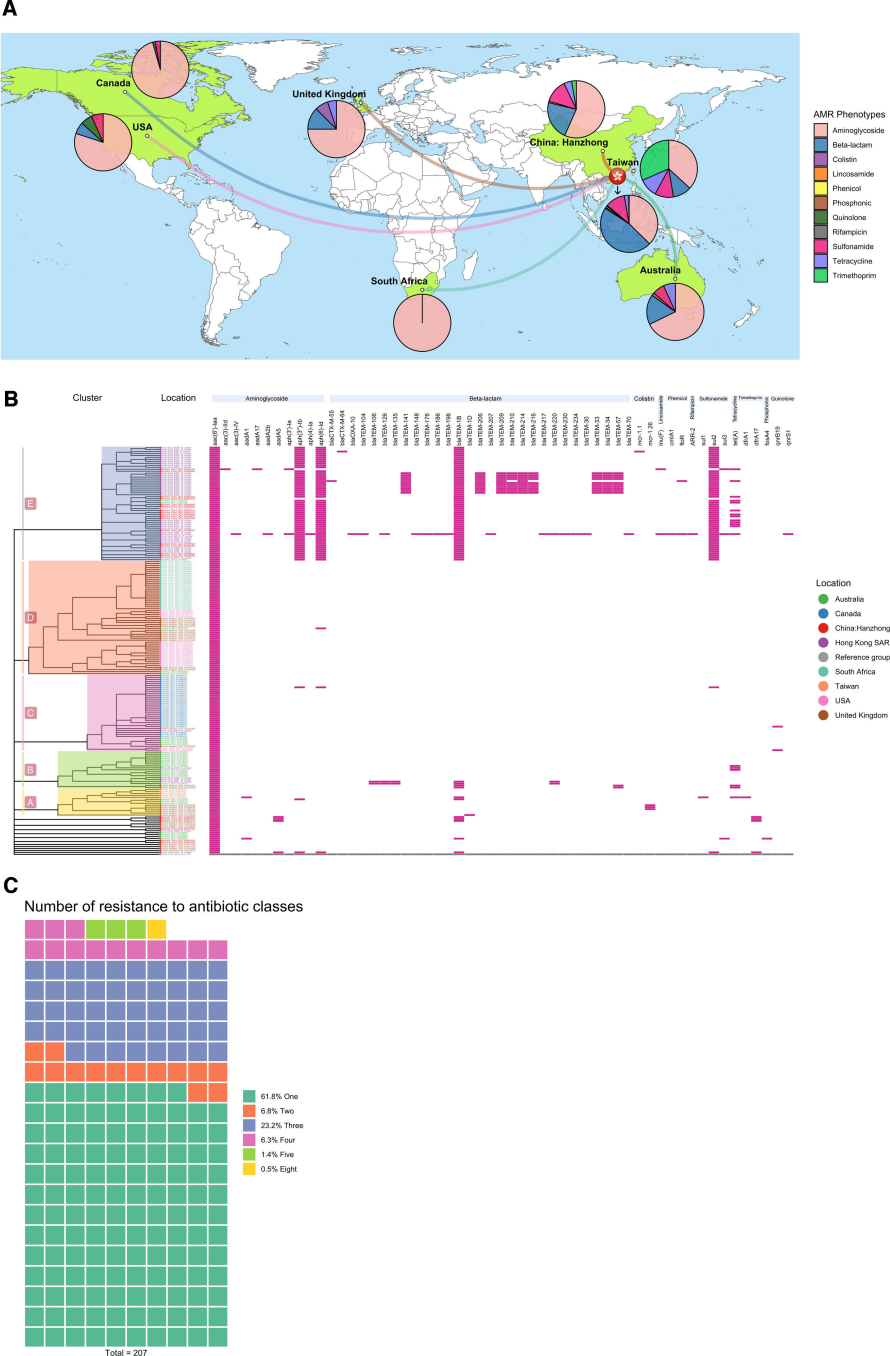
Antimicrobial resistance profile. (A) World map showing the distribution of AMR phenotypes and the proportion of AMR phenotypes per location is illustrated as color-coded pie charts. Map was generated from R ggmap (https://cran.r-project.org/web/packages/available_packages_by_name.html#available-packages-G). (B) Heatmap illustrating AMR genotypic and phenotypic profile with gene presence (pink) and absence (white). (C) Number of resistance antimicrobial classes. AMR = antimicrobial resistance; SNP = single nucleotide polymorphism.

We identified 55 distinct antibiotic resistance genes (ARGs) conferring AMR to 11 antibiotic classes (aminoglycoside, beta-lactam, colistin, lincosamide, phenicol, rifampicin, sulfonamide, tetracycline, trimethoprim, phosphonic, and quinolone). (Fig. 3A; Table S6). Hong Kong, Taiwan, mainland China, and Australia demonstrated a high diversity of resistant antibiotic classes in contrast to other regions or countries. Canada and the United States of America appeared to have similar AMR patterns. South Africa interestingly had the lowest AMR diversity exhibiting resistance only to aminoglycoside. The phylogenetic heatmap depicting the “resistome” displayed geographical variations in ARG densities across different antibiotic classes within various phylogenetic clusters (Fig. 3B). For example, *bla*_TEM_ and *sul2* genes were predominantly found in isolates from cluster E, which mainly consisted of samples from Hong Kong and mainland China. Conversely, clusters A and B, which encompassed a more diverse set of countries or regions, exhibited a low density but widespread distribution of ARGs, such as *mcr, tet, dfrA*, and *aph(3’). qnr*S1, a plasmid-associated resistance gene, was observed in one isolate from Hong Kong. We observed a total of 31.4% (65/207) MDR *S*. Enteritidis strains (Fig. 3C). The majority of them carried distinct genotypic patterns *aac(6′)-Iaa-aph(3″)-Ib-aph (6)-Id-bla*_TEM-1B_*-sul2* (50.8%, 33/65) and *aac(6′)-Iaa-aph(3″)-Ib-aph (6)-Id-bla*_TEM-1B_*-sul2-tet(A)* (15.4%, 10/65), conferring resistance to classes of aminoglycoside, beta-lactam, sulphonamide, and tetracycline.

### Distribution of virulence-associated determinants of *S*. Enteritidis

**Fig 4:**
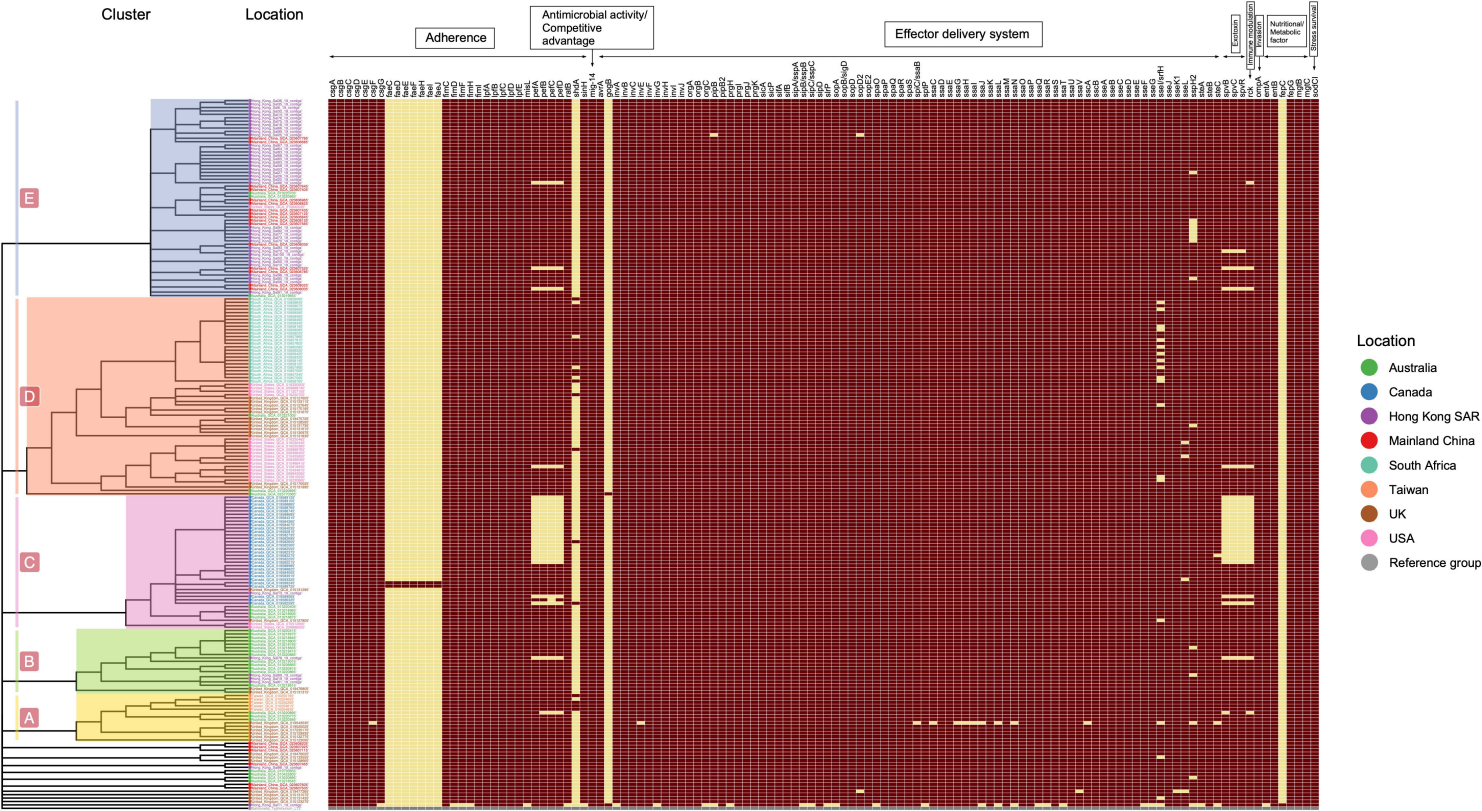
Heatmap distribution of virulent factors. The pattern virulence factor presence (chocolate) or absence (yellow) is shown for each isolate.

The genomes contained a total of 122 unique virulence-associated determinants. The most prevalent category was the effector delivery system (*n* = 77), which was primarily the type III secretion system (T3SS) encoded by *Salmonella* pathogenicity island −1 (SPI-1) (46.8%, 36/77) and −2 (SPI-2) (53.2%, 41/77) (Fig. 4; Table S7 and S8). SPI-1 predominantly encoded *inv(ABCEFGH)*, *spa(OPQRS)*, *prg(HIJK)*, and *sip(ABCD)*. SPI-2 was predominated by *ssa* genes that encode T3SS2 effector proteins to facilitate the replication of intracellular bacteria within membrane-bound *Salmonella*-containing vacuoles. Among fimbrial adherence determinants, we observed two universal gene clusters: *csg* operons and *fim* operons. *csg* operons, including *csg (ABCEFG),* encoded for thin aggregative fimbriae (Agf), which were responsible for bacterial cell adhesion to the villi of enterocytes. *fim* operons, including *fim(ICDFH)*, encoded for type I fimbriae to mediate T3SS1-independent uptake in eukaryotic cells.

### Distribution of plasmid in *S.* Enteriditis

**Fig 5:**
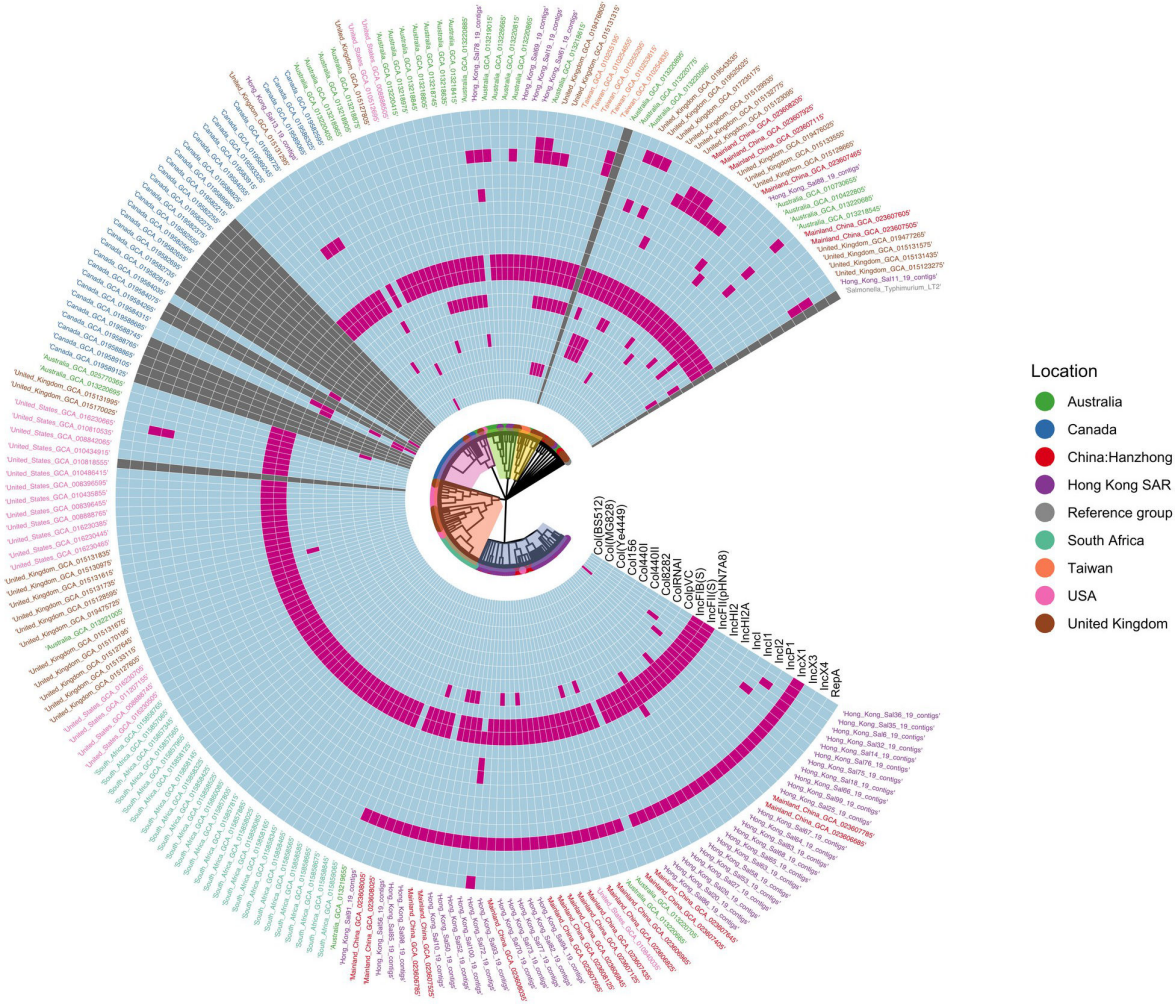
Distribution of plasmid replicon markers. The maximum-likelihood phylogeny is surrounded by colored rings representing the presence (pink) or absence (light blue) of plasmid replicon markers. Gray signifies that plasmid replicon markers were not found in *S.* Enteritidis.

Plasmid profiling revealed 22 plasmid replicon markers in 90.8% (188/207) of the isolates, including Inc groups, Col groups, and RepA. Each isolate contained a minimum of one and a maximum of six plasmid replicon markers (Fig. 5; Table S9). Most isolates carried both IncFIB(S) (94.7%, 178/188) and IncFII(S) (95.2%, 179/188) replicons. IncX1, prevalent in Cluster E, was detected in 39.9% (75/188) of isolates, including 41 from Hong Kong, 18 from mainland China, 1 from Taiwan, and 15 from the West. Notably, isolate Hong_Kong_Sal65_19_contigs exclusively harbored replicon IncFII(pHN7A8), while isolate Hong_Kong_Sal72_19_contigs was the only one to carry replicons IncHI2, IncHI2A, and RepA.

### Phylogeographic analysis of *S*. Enteritidis inferred key migration events between Hong Kong and mainland China, Australia, and Canada

**Fig 6:**
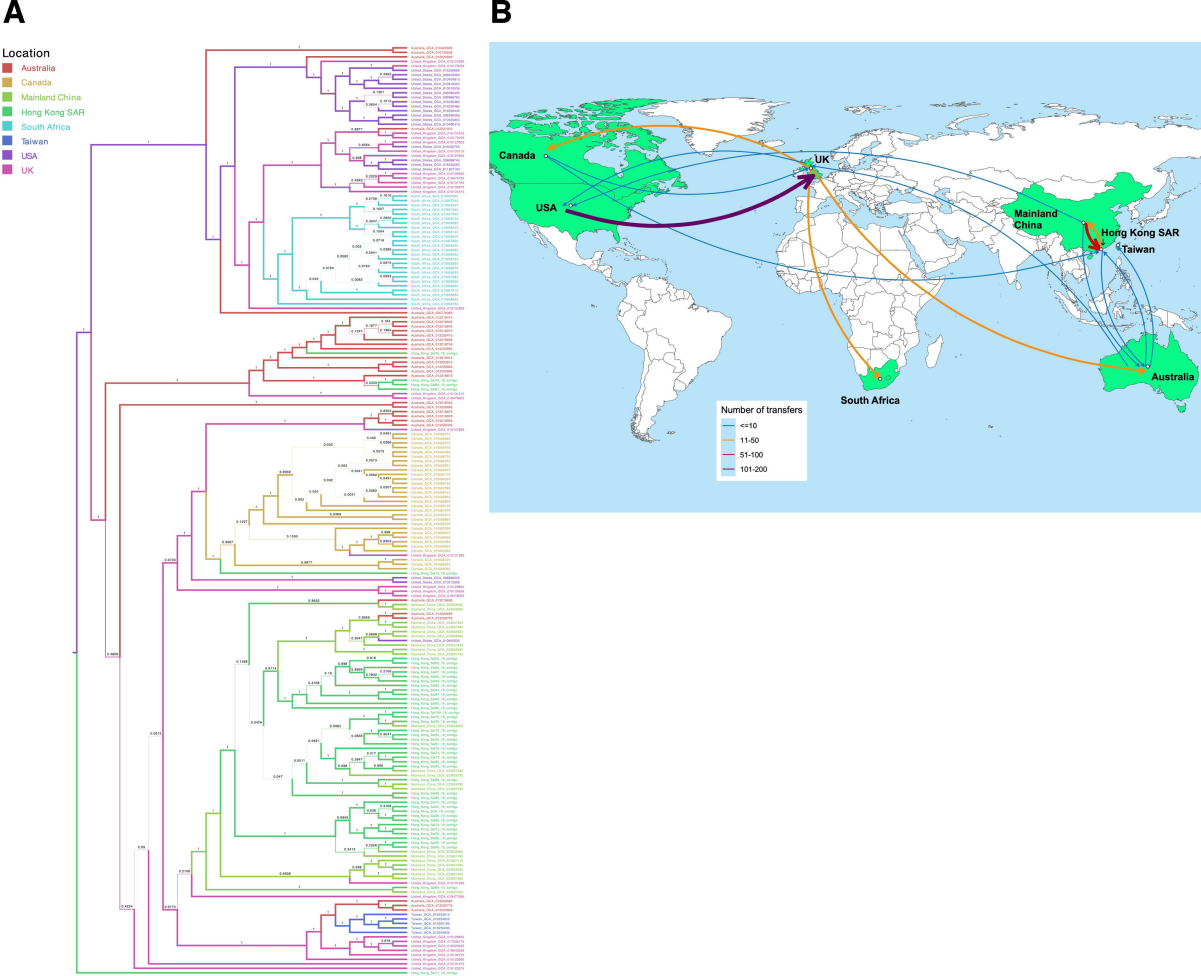
Inferred transmission dynamics of *S*. Enteritidis. (A) Bayesian maximum clade credibility, where the color of the internodal branches represents the predicted geographical origin, and the branch labels indicate the posterior probability values. (B) Major geographical transfers of *S*. Enteritidis isolates inferred from the Bayesian phylogenetic tree. The thickness of each arrow indicates the relative number of transfers between the regions or countries. Map was generated from R ggmap (https://cran.r-project.org/web/packages/available_packages_by_name.html#available-packages-G).

In this cross-sectional study, a Bayesian phylogeographic inference analysis was performed to investigate the spatial relationship of *S*. Enteritidis among countries or regions. The inferred *S*. Enteritidis migration events between these locations represent the geographical connections of *S*. Enteritidis. The directionality of migration is suggested by a maximum-clade credibility tree in Fig. 6A; Table S10. In general, *S*. Enteritidis isolates in the studied countries or regions were genetically linked through hypothetical migration events. According to the Bayes Factor (BF), the highest relative migration rate was observed between mainland China and Hong Kong (relativeGeoRates mean ± standard error = 2.93 ± .07, BF = 1285.5) (Table S10a). Statistically significant migration events inferred from the maximum-clade credibility tree are depicted as a map in Fig. 6B. The majority of isolates in Hong Kong can be primarily traced back to mainland China, with some traceable to Canada (0.61 ± 0.03, BF = 6.9) and Australia (1.02 ± 0.04, BF = 4.2). Australian isolates could be traced back to the United Kingdom (1.54 ± 0.04, BF = 426.7), and further from the United Kingdom to the United States of America (1.18 ± 0.03, BF = 1285.5). Isolates in mainland China, on the other hand, could not be ancestrally traced to other countries or regions, but there were some hypothetical migration events towards Australia (1.16 ± 0.04, BF = 3.1) and the United States of America (0.72 ± 0.04, BF = 3.2). While other countries or regions had migration events with at least two locations, isolates in South Africa and Taiwan could only be traced to one single location, with migration events from the United Kingdom to South Africa (0.61 ± 0.04, BF = 3.3) and from Australia to Taiwan (0.69 ± 0.04, BF = 4.6), respectively.

## DISCUSSION

*S*. Enteritidis was reported to be the most common nontyphoidal *Salmonella* serotype in Hong Kong (25–28, 30–34). Our findings identified three significant hypothetical migration routes of *S*. Enteritidis from mainland China, Australia, and Canada into Hong Kong with statistical evidence. Under the principle of “One country, two systems,” Hong Kong and mainland China are closely related to frequent cross-boundary travel, and Hong Kong heavily relies on mainland China for food supply. As *S*. Enteritidis has broad host ranges that can be transmitted via food or travelers, the close relationship between Hong Kong and mainland China in many aspects may contribute to the similarity of *S*. Enteritidis at the genetic level. Our findings were consistent with a previous study (61), which noted a unique phylogenetic pattern of *S*. Enteritidis in mainland China at a global level, as indicated by the lack of hypothesized migration events toward mainland China in our study. Migrations from Australia and Canada, both significant food suppliers to Hong Kong, could be possibly connected to international trade and travel. Interestingly, Australian *S*. Enteritidis isolates could be traced back to the United Kingdom, which is consistent with one study finding showing that Australian *S*. Enteritidis may originate from other countries like the United Kingdom through international travel (62). The United Kingdom, as a mid-point, can be further ancestrally traced to the United States of America, supported by returning traveler data (63). While other countries or regions are also parts of the food supply chain to Hong Kong, their *S*. Enteritidis were not statistically connected to Hong Kong, suggesting other factors might play a significant role that need further investigation. The finding provides insight into the timing and location of AMR emergence, as well as the phylogeographical spread of *S*. Enteritidis. This enables the quantification of the effects of AMR burden and offers new evidence of implementing effective AMR containment and food safety interventions at the international level.

Our analysis demonstrates that mainland China is an important transmission source for AMR in *S*. Enteritidis to Hong Kong, which can be linked to poor hygienic practices and food contamination (12, 64, 65). China is one of the largest animal food production economies, and 70%–80% of bacterial foodborne infections are caused by *Salmonella* (6, 66). A global phylodynamic analysis of *S*. Enteritidis, which includes over 30,000 genomes from 98 countries from 1949 to 2020, along with the international trade of live poultry from the 1980s to late 2010, suggests that the international trade of poultry breeding stocks and production systems could be contributing factors for transmission (67). Changes in animal production systems in a region, such as increased consumption of antimicrobials, may create ecological niches conducive to the thriving of *S.* Enteritidis with certain suitable genetic and phenotypic traits, which poses a great challenge for tracking the transmission source of human salmonellosis.

When introduced to a new environment, susceptible *S*. Enteritidis quickly adapts, acquires new resistance, and develops MDR across a specific geographical region. More than 30% of MDR strains were observed mostly from mainland China and Hong Kong, with the most prevalent ARGs being extended-spectrum beta-lactamase genes. The most common resistance genes were *aac(6′)-Iaa, aph(3″)-Ib, aph (6)-Id*, *bla*_TEM-1B_, *sul2* and *aac(6′)-Iaa, aph(3″)-Ib, aph (6)-Id*, *bla*_TEM-141_, *bla*_TEM-1B_, *bla*_TEM-206_, *bla*_TEM-209_, *bla*_TEM-210_, *bla*_TEM-214_, *bla*_TEM-216_, *bla*_TEM-33_, *bla*_TEM-34_, *bla*_TEM-57_, and *sul2.* Surprisingly, South Africa, a country that constitutes a significant number of *Salmonella* morbidity and mortality cases, had the least number of resistance genes, and no MDR strains were captured. This finding is not consistent with the annual global estimation of salmonellosis, which was reported to be higher in countries with poor nutrition and limited healthcare access (3, 68–70). This discrepancy could be due to the limited availability of South Africa genome information (only 25 South Africa isolates obtained from human stool were publicly available during the study period) and the limited use of WGS for routine surveillance and outbreak tracking in resource-limited settings; thus, the analysis reported here should be considered conservative. Moreover, the studied isolates from South Africa were from two concurrent outbreaks in 2018 (71) and may not represent the locally circulated MDR *S*. Enteritidis.

Extensive use of antibiotics in livestock production has been documented, with China and South Africa being one of the largest consumers of veterinary antibiotics globally (72). In fact, in China, the resistance rate to the front-line drugs for salmonellosis is higher than in other regions. Although China has implemented stricter regulations to control antibiotic consumption in livestock production (73), a Chinese survey on 88 chicken farms in northwestern China found that 75% of farmers used prohibited antibiotics, while 14.8% continued consumption of antibiotics during the withdrawal period (74). Another important study by Lai et al. (75), examining resistance in *Salmonella* in the Shandong Province of China from 2009 to 2012, detected significant resistance to nalidixic acid (95.9%), ampicillin (72.3%), ciprofloxacin (41.5%), and ceftiofur (42.2%) (75). In contrast to Africa, there is no significant progress in the implementation of antibiotic stewardship and surveillance programs in human and animal systems (70). Consequently, the increasing AMR crisis is worsened by the widespread indiscriminate use of antibiotics, poor clinical care, inadequate regulations on antibiotics, and a lack of regional surveillance on AMR and antimicrobial use (76–78). According to a 2016 report by the World Organization for Animal Health (79), it was found that antimicrobial growth promoters were authorized for use in 15% of African countries. However, the findings from the 2010–2012 survey on antibiotic consumption in southwestern Nigeria increased by 40.4% within the 3 years, with the majority being tetracyclines (33.6%), fluoroquinolones (26.5%), and beta-lactams/aminoglycosides (20.4%) (80). Such inappropriate practices can lead to serious consequences of antimicrobial residues in food animals, which have been reported in several African countries including Ghana, Kenya, Nigeria, and South Africa (81). The differences in antibiotic governance and consumption practices may contribute to the observed discrepancies in the spread of ARG prevalence between these regions. Understanding these dynamics underscores the importance of prudent antibiotic use in agriculture to combat the resistance spread.

Despite insightful details about AMR and inferred phylogeographic analysis, some limitations should be acknowledged to better comprehend the key results proclaimed. The present study assesses *S.* Enteritidis isolated from human samples but is limited in its timeframe, study location, and sampling types as per our search strategy. This study also under-represents sequences from certain regions/countries in the public database, for example, there is an absence of MDR *S.* Enteritidis from South Africa where salmonellosis is endemic. The cross-sectional nature of this study means that the information was obtained over a limited timeframe and might not fully represent the situation. In certain areas with denser sampling, such as mainland China, most isolates were derived from a small number of surveillance sites, i.e., Hanzhong, a single sampling site in Northwest China. As a result, these isolates might not be representative of the true circulating strains in mainland China. During our search on the NCBI databases, we also encountered some search results with either absent associated metadata or even assemblies. Extra efforts were required to trace the sequence bio-numbers for clinical data, and some without assemblies had to be excluded from the final analysis. This might significantly underestimate the true risk assessment of AMR and the migration events of imported *S*. Enteritidis in the global context.

### Conclusion

This analysis provided the first evidence of phylogeographic analysis of drug-resistant *S.* Enteritidis, showing cross-border transmission of strains and resistance expansion. The statistically significant migration events of AMR and drug-resistant strains from other continents into Hong Kong highlighted the importance of *S*. Enteritidis control in international food trading to safeguard human health and food safety. The persistence of MDR strains specifically in mainland China and Hong Kong necessitated effective controlling and monitoring of antibiotic use and animal farming practices. The international spread of AMR should be considered a global rather than a local problem. Future phylogenomic studies should include a larger number of clinical *S*. Enteritidis genomes, span a broader sampling timeframe, and incorporate more sequences from various continents in order to improve the investigation of phylogenies and transmission patterns to provide evidence-based information to support global efforts mitigating the resistance spread.

## Data Availability

Detailed methods, results, and additional data are available in this manuscript and the associated appendix. The custom Python and R scripts, along with the input files used in this analysis, can be freely obtained from https://github.com/YipHoChan/Salmonella-Enteritidis-HK.

## SUPPLEMENTAL MATERIAL

Supplemental tables spectrum.02292-24-s0001.xlsx Tables S1 to S11.

ASM does not own the copyrights to Supplemental Material that may be linked to, or accessed through, an article. The authors have granted ASM a non-exclusive, world-wide license to publish the Supplemental Material files. Please contact the corresponding author directly for reuse.
